# Therapeutic Potential of EVs: Targeting Cardiovascular Diseases

**DOI:** 10.3390/biomedicines11071907

**Published:** 2023-07-06

**Authors:** Javier Laura Francés, Christina Pagiatakis, Vittoria Di Mauro, Montserrat Climent

**Affiliations:** 1 IRCCS Humanitas Research Hospital, 20089 Rozzano, Italy; javier.laurafrances@humanitasresearch.it (J.L.F.); or christina.pagiatakis@uninsubria.it (C.P.); vid4010@med.cornell.edu (V.D.M.); 2 Department of Biotechnology and Life Sciences, University of Insubria, 21100 Varese, Italy; 3 Veneto Institute of Molecular Medicine, Via Orus 2, 35129 Padova, Italy; 4 Department of Pathology and Laboratory Medicine, Cardiovascular Research Institute, Brain and Mind Research Institute, Weill Cornell Medicine, 1300 York Avenue, New York, NY 10065, USA

**Keywords:** extracellular vesicles, exosomes, EVs, cardiovascular diseases, myocardial infarction, diabetic cardiomyopathy, septic-induced cardiomyopathy

## Abstract

Due to their different biological functions, extracellular vesicles (EVs) have great potential from a therapeutic point of view. They are released by all cell types, carrying and delivering different kinds of biologically functional cargo. Under pathological events, cells can increase their secretion of EVs and can release different amounts of cargo, thus making EVs great biomarkers as indicators of pathological progression. Moreover, EVs are also known to be able to transport and deliver cargo to different recipient cells, having an important role in cellular communication. Interestingly, EVs have recently been explored as biological alternatives for the delivery of therapeutics, being considered natural drug delivery carriers. Because cardiovascular disorders (CVDs) are the leading cause of death worldwide, in this review, we will discuss the up-to-date knowledge regarding the biophysical properties and biological components of EVs, focusing on myocardial infarction, diabetic cardiomyopathy, and sepsis-induced cardiomyopathy, three very different types of CVDs.

## 1. Introduction

Cardiovascular disorders (CVDs) encompass a group of pathologies that affect the heart and blood vessels. Despite significant advances in cardiovascular research that have increased patient survival, according to the American Heart Association, only in 2020, approximately 19.1 million deaths were attributed to CVDs globally, with a socio-economic burden that in the United States alone was estimated to cost around USD 219 billion each year [1]. The absence of good biomarkers for the early detection of CVDs, the complex procedures and post-operative complications of cardiac surgery, and the shortage of cardiovascular drug innovations in comparison to other fields, like oncology and neurology, render CVDs the leading cause of death worldwide [2]. Therefore, novel strategies and methods are urgently needed for CVD therapy. The cardiovascular system is a highly complex, well-organized structure of heterogeneous cell populations represented by cardiomyocytes (CMs) and non-muscle cells, such as fibroblasts (FBs), smooth muscle cells (SMCs), endothelial cells (ECs), neuronal cells, and inflammatory cells [3]. It is well known that the crosstalk and intercellular communication between these cells allow the cardiovascular system to function as an integrated unit, with proper myocardial contractility, sufficient perfusion, balanced myocardial extracellular stiffness, and controlled functioning of the immune system [4]. However, when disturbed, this dynamic interplay triggers or exacerbates maladaptive mechanisms, including cardiac hypertrophy, fibrosis, and chronic inflammation, critical hallmarks for the onset of cardiovascular disorders [5]. Given the complex and heterogeneous nature of the pathophysiology of CVDs, therapeutic approaches based on molecular mechanisms are more challenging to come by. Although the cellular and molecular mechanisms pertaining to myocardial infarction (MI) have been widely studied, especially in the context of cellular communication and EVs, diabetic cardiomyopathy (DCM) and septic-induced cardiomyopathy (SIC) remain unexplored. Therefore, we aimed to highlight the known mechanisms of EVs in MI, making parallels to two less-studied CVDs, namely DCM and SIC.

Myocardial infarction, leading to heart failure, is one of the leading causes of morbidity and mortality worldwide. At the pathophysiological level, MI is the result of cardiac ischemia following coronary artery occlusion. At the cellular level, the onset of cardiac ischemia results in a hypoxic state, which not only damages CMs but activates pro-inflammatory responses and FBs, leading to fibrosis. As a result of this, MI results in irreversible myocardial tissue damage, leading to cardiac remodeling and, eventually, heart failure [6]. Ischemia, which is the result of arterial obstruction, results in a decrease in blood and oxygen supply to the heart, resulting in a decrease in cardiac function. More specifically, three phases of MI have been previously described as a result of the hypoxic state of the heart. The first phase, which occurs between the first three days, results in left ventricular remodeling due to an inflammatory response. Over this time frame, hypoxia conditions exacerbate necrosis and apoptosis pathways in the cell, resulting in the release of free radicals and chemokines, including reactive oxygen species (ROS), damage-associated molecular patterns (DAMPs), tumor necrosis factor (TNF), interleukin 1 (IL-1), and interleukin 10 (IL-10). In the second phase, a proliferative state has been observed, whereby the chemokines and matrix metalloproteinases (MMPs) secreted by FBs promote the infiltration of leukocytes and the expression of pro-inflammatory cytokines that recruit pro-inflammatory FBs. The activation of myofibroblasts results in secretion of collagen III, promoting left-ventricular wall remodeling and the deposition of scar tissue. Such processes result in CM damage and loss, a major contributor of eventual heart failure. In the final “maturation” phase, which can be acute, left ventricular remodeling has been Shown to contribute to diastolic dysfunction, cardiac arrhythmias, and cardiac hypertrophy [7].

Diabetes mellitus is a metabolic pathology affecting more than 450 million people and is characterized by hyperglycemia. Diabetic cardiomyopathy (DCM) is one of the major causes of death in patients with diabetes and is defined as a ventricular dysfunction that can lead to heart failure in diabetic patients without the presence of other comorbidities, such as hypertension or coronary artery disease [8,9,10]. Diabetes causes severe abnormalities in the overall structure and function of the heart, affecting many mechanisms. Overall oxidative stress, as well as mitochondrial dysfunction, are observed in diabetic hearts. Oxidative stress is induced in many circumstances during pathologic DCM. For example, hyperglycemia is able to alter myocardial stiffness by modulating structural proteins through advanced glycation end products (AGEs), which accumulate in the vessel wall, thus being directly involved in vascular pathologies such as diabetes. AGEs are involved in the activation of NF-kB as well as in ROS production by modulating NO activity in ECs. Oxidative stress, like ER stress, also leads to inflammation during DCM, regulating cell adhesion molecules, immune cell infiltration, and, in turn, the increased expression of pro-inflammatory cytokines. Impaired cardiac contractility, caused by the aberrant handling of calcium, is another aspect during DCM due to decreased GLUT4 expression associated with fatty acid accumulation and oxidation. All these conditions are generally linked to increasing levels of apoptosis and necrosis. Autophagy, which is necessary for normal cellular function, is also aberrantly regulated in cardiac hypertrophy during DCM. Another important mechanism affected during DCM is fibrosis. Increased levels of collagen deposition as well as the deregulated activity of MMPs, cause increased connective tissue within diabetic hearts [11,12,13,14,15,16].

Sepsis is a systemic inflammatory condition caused by an exaggerated host response to infection. Septic shock is the most severe complication of sepsis, which occurs when affected patients display hypotension with consequent organ failure and elevated serum lactate concentrations [17]. So far, despite advances in antibiotic therapy and the critical care management of sepsis, multi-organ failure with morbidity and mortality continues to remain high [17]. Indeed, sepsis is one of the leading causes of death, affecting about 49 million people worldwide [18], and is associated with a mortality rate of 30% [19,20]. During sepsis, the heart is one of the major target organs. Septic-induced cardiomyopathy (SIC) is an acute cardiac disorder caused by sepsis, occurring in up to 50–60% of septic patients [17,21]. Although SIC is reversible at the early stage of sepsis, with survivors showing a complete recovery within 7–10 days, it has been reported to be associated with an unacceptably high mortality rate, resulting in death in 70–90% of cases [22]. SIC is generally defined as a decrease in left ventricular ejection fraction (LVEF) and ventricular dilatation during sepsis with the exclusion of other conditions, leading to cardiac dysfunction [23], although there is no gold diagnostic standard for SIC to date. The pathogenesis of SIC remains poorly understood, and the recommended clinical strategy for SIC patients is currently only symptomatic, and no specific treatments are available [24].

In the above-mentioned CVDs, as with many other pathologies, cellular communication could be a potential mechanism to identify new therapeutic targets for the development of new approaches and improvement of clinical outcomes and diagnosis. In the context of intercellular communication, a wide variety of signaling processes may be involved, among which cellular secretion and the uptake of extracellular vesicles (EVs) have been shown to play a pivotal role [25]. EVs are membrane-bound particles released by all types of cells into the extracellular space and were initially described as “junk material” with no functional significance or potential involvement in the clearance of damaged cellular components [3]. However, in the last decades, a plethora of studies reversed this trend by demonstrating their effective role in intercellular signaling due to their ability to transfer biomolecules, including miRNA, proteins, enzymes, cell surface receptors, growth factors, cytokines, and lipids that, in turn, can modulate target cell biology and function [1]. Furthermore, additional studies aimed at unraveling the mechanisms of biogenesis, molecular composition, and physiological and pathological functions of EVs led to the identification of major subtypes of vesicles, their association with pathological conditions, and potential therapeutic applications [26]. Importantly, several efforts have focused on properly defining EVs in order to guarantee that the effects obtained in the studies were derived exclusively from EVs rather than from other materials obtained during EV isolation. For this reason, MISEV2018 was developed to provide guidelines for basic EV characterization [27].

It is well-accepted that EVs hold great potential as source biomarkers, therapeutic targets, and vehicles for therapeutic molecules. Thus, this review will briefly discuss the current knowledge about the biophysical properties and biological components of EVs. We will describe the functions of EVs in the CVD field, focusing on myocardial infarction, diabetic cardiomyopathy, and sepsis-induced cardiomyopathy, highlighting the latest advances of EVs as prognostic and diagnostic biomarkers, their critical role at the cell-to-cell communication level and, finally, their potential use as therapeutic agents. Ultimately, we will introduce the specific application of EVs as a novel drug delivery tool and its application in CVD therapy.

## 2. Extracellular Vesicles: Exosomes and Microvesicles

Extracellular vesicles (EVs) are a heterogeneous group of spheroid or cup-shaped membranous structures of various sizes released by living cells in extracellular spaces [28]. Although the classification of EVs is continually evolving [25], they are generally divided in three major categories: apoptotic bodies, ectosomes, or microvesicles (MVs) and exosomes (EXOs). Here we will focus on MVs and EXOs.

Exosomes are the smallest class of EVs, with a size of 40–160 nanometers in diameter (averaging 100 nanometers); they are actively synthesized by the endolysosomal pathway and secreted into the intercellular space or into the systemic circulation. Briefly, an initial inward budding of the plasma membrane, which includes the cell-surface proteins and soluble proteins associated with the extracellular milieu, allows for the formation of early sorting endosomes (ESE). From this subcompartment, the cargo is sorted into one of three destinations: recycling, degradation, or secretion. These routes require a first step of maturation of the ESE into late sorting exosomes (LSEs). Subsequently, a second invagination step involving the plasma membrane in the LSE leads to the generation and the formation of intracellular multivesicular bodies (MVBs) containing intraluminal vesicles (ILVs). At this point, these MVBs can either fuse with lysosomes (L) to generate endolysosomes (EL) or with the plasma membrane to release ILVs to the milieu as exosomes [29,30]. The process of sorting cargo within exosomes involves the endosomal sorting complexes required for transport (ESCRT) and tetraspanin- and lipid-dependent mechanisms [31]. The ESCRT complex is a cytoplasmic multi-subunit system made up of four components (0, I, II, and III) that are essential for facilitating MVB formation, vesicle budding, and protein cargo sorting [31]. As a first step, ubiquitinated cargos are recognized and sequestered to specific domains of the endosomal membrane via ubiquitin-binding subunits of ESCRT-0. Subsequently, ESCRT-0 recruits ESCRT-I, which, along with ESCRT-II, promotes endosomal inward budding around clusters of ubiquitinated proteins [32]. Ultimately, following the cleavage of the buds to form ILVs, the ESCRT-III complex separates from the MVB membrane with energy supplied by the sorting protein Vps4 [33]. Interestingly, different studies have also suggested an ESCRT-independent pathway in exosome biogenesis and cargo loading, which involves lipids. The first mechanism described is lipid-mediated biogenesis. Raft-based microdomains, which are present on the plasma membrane of endosomal compartments, are enriched in sphingolipids that represent substrates for the neutral sphingomyelinase (nSMase), a lipid-metabolizing enzyme that converts sphingolipids to ceramides: this conversion, in turn, induces the proper curvature of inward budding for the formation of ILVs [29,34]. On the other hand, tetraspanin-mediated biogenesis involves another lipid-dependent process involving tetraspanin proteins (CD63, CD81, CD82, or CD9). This mechanism plays a role in exosome biogenesis and protein loading. Due to their cone-shaped conformation and their ability to cluster into microdomains (tetraspanins-enriched microdomains, TEMs), these proteins induce the inward budding of the late endosomal membrane and promote exosome formation [35]. Since tetraspanins are highly enriched within exosomes, they have been specifically used as exosomal biomarkers over the last several years [35].

Microvesicles (MVs) are large EVs, which range between 40 and 1000 nm in diameter, and form through outward budding at the plasma membrane. MVs are able to carry and transfer bioactive molecules (proteins, DNA, mRNA, and miRNAs) to the extracellular environment. MVs can interact with the recipient cell by activating signaling pathways through receptor interaction or by being engulfed through endocytosis or fusion. Indeed, MVs have been involved in cellular communication due to their wide impact on different biological processes, including inflammation, coagulation, stem cell development, and tumor progression [36]. The biogenesis of MVs involves cytoskeleton rearrangements, which promote the clustering of lipid and transmembrane proteins, leading to the direct outward budding of the membrane and ultimately releasing the MVs into the extracellular environment. The GTP-binding protein, ADP-ribosylation factor 6 (ARF6), a key regulator of MV shedding, activates the extracellular signal-regulated kinase (ERK) cascade, which leads to the downstream activation of myosin light chain (MLC), promoting the contraction of the actin cytoskeleton. Interestingly, ARF6 has also been involved as a regulator of the selective recruitment of cargo into MVs [37,38,39]. Moreover, the small GTP protein, Ras homolog family member A (RhoA), which is well-known for regulating the rearrangement of the actin cytoskeleton, is involved in actin remodeling and is, therefore, essential for effective vesicle fission.

## 3. Different Roles of Extracellular Vesicles in Cardiovascular Research and Diagnosis

### 3.1. Extracellular Vesicles Used as Biomarkers

Biomarkers are usually used as indicators to correlate pathogenic levels of certain processes in order to improve the early detection of disease, evaluate the progression of pathology, follow the development of drug treatment, predict a response to therapy, identify new therapeutic targets, or even a clinical endpoint in clinical trials [40]. Circulating biomarkers from body fluids became the most useful noninvasive way of identifying and detecting interesting biomarkers for different pathologies, including CVDs. Indeed, several biomarkers based on proteins, lipids, carbohydrates, and nucleic acids have been described in CVDs [41]. For example, high levels of circulating cardiac-related proteins, such as cardiac troponin I (cTnI) and T (cTnT), which are released by necrotic CMs, are found to increase in patients with severe heart failure [42,43]. MMP3, osteopontin, and the N-terminal propeptide of B-type natriuretic peptide (NT-proBNP) are considered biomarkers in diabetic patients [40,44]. Moreover, noncoding RNAs (ncRNAs) [45,46] have been shown to be useful biomarkers since several circulating ncRNAs have been associated with different CVDs. Regardless, further characterization needs to be carried out to identify and correlate unique ncRNAs to specific pathologies without overlapping with other diseases. Interestingly, SNPs within ncRNAs have also been associated with CVDs [47]. More recently, EVs have been gaining much interest, not only being able to serve as communication vehicles that carry and transport different functional molecules to distant sites or by acting as “active molecules” themselves but also as biomarkers, being released and loaded with cellular material that might be specifically dysregulated in pathophysiological conditions [26,48]. In physiological conditions, they are found in all body fluids, demonstrating a role not only during the development of pathologies but in the maintenance of the homeostatic state of cells and organs. Importantly, when compared to most biological biomarkers, EVs carry specific characteristics from their cell of origin, harboring information about healthy or diseased cell states [48,49]. Moreover, the content within the EVs remains stable and is protected from degradation due to their lipid bilayer membrane. Therefore, EVs are considered excellent biomarkers [50].

In this section, we aim to summarize those studies where EVs have been identified as biomarkers in myocardial infarction, diabetic cardiomyopathy, and septic-cardiomyopathy, highlighting the potential of EVs as targets for the detection of deregulated levels of specific molecules during CVD.

#### 3.1.1. Myocardial Infarction

The inherent capability of EVs to modulate intercellular communication in homeostasis and disease renders them a prime candidate for a biomarker. As previously mentioned, EVs carry bioactive cargo, including RNA and protein capable of activating a myriad of signaling pathways involved in cellular regulation and homeostasis and, very importantly, under aberrant conditions, during various pathophysiologies.

Circulating EVs are of extreme importance in the cardiovascular field, specifically as diagnostic biomarkers. Interestingly, platelet-derived EV release is increased in plasma after MI and also after exposure to modified lipoproteins. Moreover, the increase in circulating EVs can be detected in short time frames post-pathological stimulus: the amount of EVs is already detectable one hour after MI, with a significant increase in heart tissue 24 h post-myocardial ischemia/reperfusion (I/R). Endothelial-derived EVs increased in the plasma levels of HF patients who had a higher probability of cardiovascular events. It was also shown that in chronic HF, the number of circulating endothelial EVs increases, an increase that is correlated with increased mortality; such an observation indicates that the number of EVs might be important in assessing the severity of HF [51].

Noncoding RNAs, especially miRNAs, have been highly implicated as biomarkers and therapeutic molecules for cardiovascular disease and MI. Although many miRNAs have been described in cell-to-cell communication through small and large EVs, long noncoding RNAs (lncRNAs) have been less studied in this context. In the last several years, many lncRNAs have been described as key molecules in cardiovascular disease and heart failure through the epigenetic regulation of gene expression [52]. Moreover, lncRNA-enriched secreted EVs were present in cardiac ischemia. The lncRNA *Neat1* was shown to be a key player in FBs and CMs survival: Neat1 knockdown presented with reduced heart function post-MI [53]. In coronary artery disease (CAD), it was shown that the increased expression of *miR-126* in circulating EVs reduces the risk of major cardiovascular outcomes. Moreover, *miR-126* was shown to be a good candidate for the early detection of myocardial infarction: reduced plasma levels of EV-*miR-126* in high-risk CVD patients was negatively correlated with cTnI and NT-proBNP, suggesting *miR-126* as a potential biomarker for CVD [51]. Interestingly, studies have shown that there is an increase in miR-192, miR-194, and miR-34a expression in the circulating miRNAs of post-MI patients [51,54]. Interestingly, it was shown that serum EV-derived *miR-1915-3p*, *miR-457*, and *miR-3656* were significantly decreased in MI patients in comparison with patients with stable CAD [55]. Furthermore, *miR-1* and *miR-208* were found to be increased in the urine and serum of rats post-acute MI through exosome release from the damaged myocardium. Therefore, circulating microRNAs prove to be strong candidates for prognostic and diagnostic tools post-MI [56,57]. Moreover, plasma-derived extracellular vesicles carrying *miR-130a* were shown to attenuate cardiac remodeling post-MI through PI3K-dependent signaling and autophagy pathway regulation and have been documented as a diagnostic tool in this context [58].

Cardiac exosomes have also been shown to play a critical role in cardiovascular disease: under stresses such as hypoxia, CMs have the capability to change the cargo in their exosomes and increase exosome release, providing yet another example of the importance of EVs as biomarkers. Under severe or prolonged ischemia, autophagy can result in cardiomyocyte cell death. Moreover, hypoxia can induce exosome secretion by FBs enriched in HIF-1α and transforming growth factor β (TGF-β): this secretion induces vascular endothelial cells to undergo endothelial to mesenchymal transition resulting in an overexpression of extracellular collagen I, collagen III, and fibronectin [59]. Cardiac exosomes and their cargo have been shown to be paramount in MI and myocardial injury. For example, CMs have been shown to secrete *miR-30a* when subject to hypoxic conditions. In such conditions, the inhibition of miR-30a release results in the maintenance of the autophagy response in CMs, providing not only a potential molecular mechanism of the hypoxic response but a powerful diagnostic tool [60]. The study of EVs in the context of intercellular signaling in MI could be key in discovering novel biomarkers for prognostic and therapeutic purposes. A recent study analyzed the protein and microRNA content from EVs: this study revealed that the levels of *miR-340* and *miR-424* decreased, whereas the levels of *miR-29b* increased in MI patients when compared to healthy controls, suggesting an important correlation between circulating EV content and prognosis [61]. Another example of a potential EV biomarker in hypoxic diseases such as MI is CD172a+. This study revealed a strong potential clinical relevance of circulating cardiac CD172a+ EVs as a more favorable diagnostic tool in aortic stenosis patients [49]. Interestingly, the EV proteomic profiling of patients suffering from MI revealed six potential proteins that could be exploited as biomarkers: these proteins are involved in lipid metabolism (APOD and APOC3), complement activation (C1Q1A and C5), and platelet activation pathways (GP1BA and PPBP). Such proteins provide yet another potential circulating biomarker for the prediction of MI and myocardial damage [62]. As an indicator of therapeutic response, platelet P2Y12 receptor inhibitors or antagonists were shown to alter the EV counts in plasma. Importantly, treatment with ticagrelor showed lower platelet- and leukocyte-derived EV levels when compared to patients using clopidogrel treatment after acute myocardial infarction, providing yet another example of the potent use of EVs as biomarkers and therapeutic targets [51].

Ceramides and phosphatidylcholines (PCs) are bioactive lipids and lipid bilayer membrane components that are critical in predicting cardiovascular outcomes. These biomolecules are an important part of the cell membrane but have also been attributed to controlling various signaling pathways, regulating the homeostatic response, and providing yet another diagnostic tool for MI and cardiovascular disease [63]. Another recent study evaluated the diagnostic potential of the lipidomic signature of circulating EVs in patients presenting with ST-segment-elevation myocardial infarction by identifying 20 sphingolipid species. Interestingly, EVs containing ceramides, dihydroceramides, and sphingomyelins increased in the MI patients correlating to hs-troponin, leukocyte count, and ejection fraction. Such results identify novel sphingolipid biomarkers in MI patients that could be utilized as prognostic tools [64] (Table 1).

#### 3.1.2. Diabetic Cardiomyopathy

Extracellular vesicles can be extremely important as biomarkers in order to evaluate many pathological characteristics, such as identifying the molecules present at different stages of pathology, identifying early detection biomarkers, or understanding the development of drug treatment. However, few studies have been conducted to demonstrate the importance of exosomal miRNAs as promising biomarkers in DCM.

DCM can be caused by myocardial stenosis, which is an aberrant accumulation of fatty infiltrates in the heart of diabetic patients [65]. For example, in these patients and in a mouse model of insulin resistance induced by a high-fat diet, levels of circulating miR-1 and miR-133 in the serum increased. Thus, in an effort to identify novel biomarkers for DCM, the authors further investigated the potential role of these miRNAs as biomarkers using an in vitro model of lipid accumulation, whereby HL1 cells were treated with lipoproteins without causing the induction of apoptosis. The authors observed increased levels of these miRNAs expressed by the CMs along with an accumulation of miRNAs within the exosomes released in the medium [66], therefore demonstrating that the levels of *miR-1* and *miR-133* in serum can be used for the prediction of myocardial steatosis in diabetic patients. Although the authors showed high expression within the exosomes released after lipid accumulation, further studies should be performed to ascertain their potential role as cardiac cargo transferred by EVs.

Another study pinpointed the role of exosomal miRNAs as biomarkers of HF with preserved ejection fraction (HFpEF) in diabetic patients. Huang et al. (2022) studied the role of miRNAs in the exosomes from the plasma of HFpEF diabetic rats and found 12 circulating miRNAs that were reduced in the heart compared to nondiabetic rats, which was associated with HF. Interestingly, only six of these were also downregulated in the isolated exosomes, and one was upregulated. Among all the miRNAs discovered, only two (*miR-30d-5p* and *miR-126-5p*) were found to be expressed at lower levels in either exosomes or cardiac tissue, reducing the overall cardiac output (abnormal diastolic function), proposing that they may play a role in the progression of HFpEF in patients with DCM [67] (Table 1).

#### 3.1.3. Sepsis-Induced Cardiomyopathy

As previously mentioned, sepsis-induced cardiomyopathy (SIC) is a prevalent complication of sepsis, causing exceptionally high death rates, and the early and specific diagnosis of the disease still remains challenging. Elevated levels of the classical cardiac-related biochemical markers B-type natriuretic peptide (BNP) and cTnT are not specific for the diagnosis of SIC. In particular, BNP has been found to correlate with the severity of the illness rather than SIC, and cTnT cannot be used as a predictor for SIC-related mortality [68]. Thus, it is mandatory to explore new and effective diagnostic and prognostic markers for assessing the status and severity of SIC [69]. In this context, EVs and their cargo may represent a valuable tool to diagnose septic cardiomyopathy as a source of novel biomarkers, although the advantages over the clinical criterion standards (troponin I/T) have still not been investigated.

An important example is represented by the work of Ye R et al., in which the authors analyzed the circulating miRNA content of neutrophil-derived extracellular vesicles in SIC patients and healthy controls [69]. In this study, the authors found that *miR-150-5p* and *miR-21-5p* were differentially expressed between SIC groups and healthy controls. Moreover, with respect to *miR-150-5p*, the authors hypothesized that its lower level in SIC neutrophil-derived EVs contributes to the worsening of this condition by preventing protective mechanisms and reducing cell apoptosis and inflammation.

Another example of the potential diagnostic role of EVs in the context of SIC is a recent study published by Hegyesi H et al. This study used an LPS-induced systemic inflammatory response syndrome (SIRS) mouse model and demonstrated an increase in CM-derived small and medium EVs containing troponin I and muscle-associated glycogen phosphorylase in the blood [70] (Table 1).

### 3.2. Extracellular Vesicles Used as Communication Molecules

Cell-to-cell communication is an essential process for maintaining cell and tissue homeostasis. Aberrant signaling and crosstalk between the various cells that comprise an organ led to the onset of pathophysiology. Such mechanisms are critical for regulating and adapting to extracellular responses and stress signals. The cardiovascular system is a complex network of cells whereby communication between the various cell types plays a pivotal role in the maintenance of cardiac homeostasis and, consequently, the onset of disease [71]. Given that the release of extracellular vesicles has been attributed to the maintenance of cardiac homeostasis, it is a growing field for the development of cell-based therapies. EVs have been shown to carry nucleic acid cargo (e.g., miRNAs) that is involved in cellular protection and cardiac repair. By utilizing this inherent phenomenon of EVs in cellular communication, many studies have focused on EV-dependent therapies. Current limitations include a variance in the cellular micro-environment and homeostatic state of the cell; however, EV release and its subsequent cargo during a stress response have been highly implicated in cardiovascular disease and cardiac repair therapies [6].

In general, EVs can be characterized by the cargo they carry and their surface biomarkers. Such surface protein biomarkers have been shown to include annexins (annexin 1, 5, 6, and 11), disintegrin and metalloproteinase domain-containing protein 10 (ADAM10), angiotensin-converting enzyme (ACE), EH domain-containing protein 4 (EHD4), major histocompatibility complex class II (MHC II), flotillin-1 (FLOT1), and heat-shock 70-kDA (HSC70/HSP73, HSP70/HSP72). Exosomes, more specifically, can be characterized by the presence of tetraspanin proteins, stress proteins (Hsc70 and Hsp90), membrane fusion proteins (Rabs and ARF6), and endosomal-sorting complex proteins (Alix and TSG101). Microvesicles have been shown to carry cargo similar to that of exosomes, including integrins, glycoproteins, and metalloproteinases. Given this, it is possible to track EVs released from specific cell types, identifying the recipient cell type [51].

Given the nature of EVs and their role in cell-to-cell communication, they have been studied in several contexts. More specifically, they have been shown to be released by almost all cell types, specifically by the cardiovascular system: blood, heart, and blood vessels. EVs have been shown to be released from blood cells (platelets, erythrocytes, and leukocytes) and regulate homeostasis, inflammation, and vascular integrity. Interestingly, many studies have shown that EVs can be released from all the major cell types of the heart, CMs [51,72] being able to be transferred to other cell types having a biological function.

Therefore, it is important to consider the role of EVs in intra- and extracellular communication between cardiomyocytes, endothelial cells, fibroblasts, hematopoietic cells, and smooth muscle cells in the context of the maintenance of cardiovascular homeostasis and their role in contributing to myocardial infarct, diabetic cardiomyopathy, and sepsis-induced cardiomyopathy.

#### 3.2.1. Myocardial Infarction

Cardiac EVs have been shown to have a specific proteomic signature, which is necessary for proper heart function. Studies have shown that EVs secreted by CMs are able to transfer nucleic acids, including noncoding RNAs. Cardiac EVs range in size from 40 to 300 nm (from exosomes to MVs) and contain caveolin-3 and flotillin-1 on their surface. Transcriptomic analysis showed that these EVs contain over 1500 different mRNA transcripts and 340 distinct DNA sequences, suggesting that cardiac EVs are necessary for maintaining cardiac homeostasis through the transfer of biological molecules that regulate gene expression. Moreover, a proteomic analysis of cardiac EVs showed that exosomes are enriched in sarcomeric and mitochondrial proteins and growth factors, further promoting the idea that they are necessary for the regulation of gene expression for the maintenance of cardiac homeostasis [73]. In order to understand the role of EV release in the myocardium post-MI, several studies have used the hypoxic condition in vivo and in vitro, which is a potent stimulator of exosome release. In vitro, cardiomyocytes exposed to hypoxia show the secretion of exosomes rich in Tnf-α, which is induced by the Hif-1α [74]. The cell-to-cell communication between CMs and ECs has been shown to be critical in maintaining cardiac homeostasis. It has been shown that under hypoxic conditions, the expression of *miR-126* and *miR-210* increases in ECs, promoting angiogenesis. The shuttling of these miRNAs to CMs post-hypoxia increases the resistance of cardiac progenitor cells to hypoxic stress through the Pi2k/Akt pathway, providing an important example of cell crosstalk in the maintenance of homeostasis through EVs [73].

Although long noncoding RNAs (lncRNAs) have been implicated in the onset of various cardiac pathologies, the mechanisms by which they are shuttled remain largely unknown. An interesting study revealed a novel pathway involving crosstalk between CMs and FBs, which is mediated by the transfer of lncRNA-enriched EVs in the context of cardiac ischemia. This study revealed two hypoxia-induced lncRNAs (*ENSMUST00000122745* and *Neat 1*) that were enriched in small EVs and large EVs, respectively. Vesicles containing these ncRNA were taken up by FBs, activating the profibrotic gene response and regulating CM survival post-MI [53]. Moreover, the circRNA *circ_0001747* was shown to be increased in hypoxic conditions. This circRNA targets *miR-199b-3p*, which is involved in alleviated hypoxia-induced CM damage [75].

Moreover, the presence of miRNAs in EVs in the heart is necessary for mediating cell-to-cell communication. A recent study showed that cardiosomal miRNAs play a critical role in the activation of myofibroblasts post-ischemic injury. In fact, this study showed that *miR-195* was significantly upregulated in cardiosomes and in fibroblasts isolated post-MI. This cardiomyocyte-specific miRNA is transferred to FBs through exosomes and is required for the activation of myofibroblasts in the maintenance of cardiac homeostasis [76]. Another recent study revealed that remote ischemic conditioning promotes cardioprotection through the regulation of circulating EVs that accumulate in the injured myocardium post-MI. An important miRNA regulating this process is *miR-144*, which promotes cardioprotection through circulating EVs, binding to the Argonaute-2 protein and targeting downstream cells [77]. Studies have previously revealed the regenerative role of EVs post-MI. More specifically, EPC-EV-derived paracrine signaling plays a critical role in angiogenesis, proliferation, and cell survival, mediating crosstalk between the different cell populations of the heart. In this context, miRNAs have been shown to be necessary for cardiac FBs activation and mesenchymal-to-endothelial cell transition. A recent study revealed that rat cardiac FBs cultured with EVs derived from hypoxia-induced EPCs produce a significant upregulation of *miR-133* and increase the expression of endothelial markers, suggesting that crosstalk between these cell types is necessary for the regulation of cardiac fibrosis [78]. Another important study identified hypoxia-inducible, endothelial EV-abundant circRNAs that mediate EC-CM communication during endogenous cardiac regeneration, emphasizing the importance of these mechanisms post-MI. Interestingly, the circRNA *Whsc1* was found to be enriched in neonatal mouse hearts, specifically in the endothelial cell population, and was increased post-hypoxia, inducing CM proliferation. This study provides further evidence of a potential therapeutic target for heart regeneration post-MI through an EV-dependent mechanism of cell communication [79].

The role of EVs as mediators of cardioprotection has become a very prominent topic of study. Interestingly, EVs from ischemic CMs were shown to protect against oxidative stress by promoting EC angiogenesis and proliferation through *miR-222* and *miR-143* [80]. HSP20 was also shown to be a cardioprotective factor transferred through EVs [81,82]. Another study also revealed that *miR-31* targets Fih1, inhibiting its expression. Exosomes from ASCs were shown to enhance angiogenesis in mouse ischemic hindlimbs and hearts through the delivery of *miR-31*, which targets Fih1 and, consequently, initiates Hif-1α transactivation under hypoxia conditions in the endothelium [83]. Hypoxia is a potent stimulator of EV secretion in cardiomyocytes. As previously mentioned, Hif-1α induces the expression of Tnf-α, increasing the amount of *miRNA-30a* in exosomes post-hypoxia. *miRNA-30a* has been shown to regulate the autophagy pathway in a paracrine fashion, and post-MI, the increase in this miRNA protects the myocardium through the downregulation of key factors regarding the autophagy pathway in endothelial cells [84]. Moreover, MSC stimulated with Ifn-γ increases exosomal production (MSC-EXO), and treatment with these exosomes could improve the anti-apoptotic ability and angiogenesis in CMs and ECs, respectively, thus preserving heart function in a mechanism mediated by the increased expression of the cardioprotective *miRNA-21*. This study provides another example of exosome-mediated cell communication that could be exploited for MI therapeutic targets [85]. The investigation of novel cell-to-cell communication pathways post-MI revealed that epicardial EVs enhanced proliferation in H9C2 cells and in primary neonatal murine cardiomyocytes in vitro. These EVs also contributed to cell-cycle re-entry at the infarct zone of neonatal hearts. Furthermore, a genome-wide analysis revealed conserved miRNAs between human and mouse epicardial-derived exosomes (*miR-30a, miR-100, miR-27a*, and *miR-30e*) [86].

Interestingly, the current strategies for the improvement of cardiac function post-MI include cell transplantation or the injection of cardiac progenitor cells (CPCs) to the infarct site. Studies showed that the transplantation of CPCs into the infarct site improved the ejection fraction, suggesting that EVs contribute to cardiac repair through the Yes-associated protein (Yap), indicating that EV secretion provides a short-term beneficial effect on cardiac recovery post-MI [87].

Moreover, exosomes released from immune cells have been shown to carry *miR-155*, repressing fibroblast proliferation and endothelial cell-dependent angiogenesis [88]. Finally, a recent study provided novel insights into the mechanism of exosome-dependent angiogenesis post-acute MI in ECs. This study demonstrated that exosomes from the peripheral serum of AMI patients promote angiogenesis via the *miR-126-3p*/TSC1/mTORC1/HIF-1α signaling pathway, providing another example of cellular communication and crosstalk post-MI [89].

**Figure 1:**
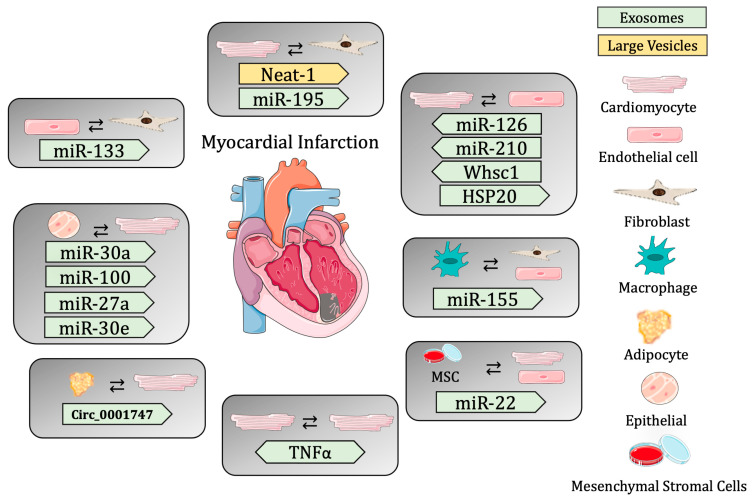
Summary of cell-to-cell communication in myocardial infarction through extracellular vesicles.

These studies reveal the importance of cell-to-cell communication, specifically mediated through extracellular vesicle release in the maintenance of cardiac homeostasis, and the importance of changes in EV cargo to the contribution of cardiac regeneration post-MI (Table 2 and Figure 1).

#### 3.2.2. Diabetic Cardiomyopathy

As mentioned above, EVs are part of a communication highway critical for cellular communication. In fact, several works have described EVs participating as communication carriers transporting different molecules from cell to cell in different physiological and pathological contexts, including DCM. Since DCM is a vascular complication of type-2 diabetes that causes damage to ECs and dysfunction to CMs within the cardiac environment, cell-to-cell communication between these two cell types is crucial for the prognosis of DCM. For example, Wang et al. demonstrated that impaired myocardial angiogenesis caused by diabetes is due to anti-angiogenic signaling sent from CMs to ECs through exosomes. Indeed, the study shows that exosomes derived from diabetic CMs have a negative effect on angiogenesis, inhibiting EC proliferation and migration. Moreover, these exosomes contained high levels of *miR-320* (anti-angiogenic) and low levels of *miR-126* (pro-angiogenic), and, in fact, the exosomes secreted by these CMs were able to transfer *miR-320* to ECs, modulating proliferation and migration during the oxidative stress in DCM [90]. In a following study, the authors further demonstrated the biological role of the exosomes released from CMs during DCM, focusing on cellular defense mechanisms, such as the activation of heat shock proteins (Hsp), which are key regulators of cellular function. The expression levels of different Hsp proteins at early (10 days) and late (3 months) time points were studied in a murine model of hyperglycemia, showing that their expression pattern is not redundant and is important for cardioprotection in diabetes. The authors focused on Hsp20, being the only Hsp regulated at both time points (i.e., increased at 10 days; decreased after 3 months). In order to understand the biological relevance of increased Hsp20 in acute hyperglycemia, the authors used a diabetic-induced mouse model overexpressing Hsp20, specifically in CMs. Indeed, the overexpression of Hsp20 ameliorated the myocardial dysfunction caused by diabetic conditions. Of note, the production and secretion of exosomes was found to increase in the CMs from Hsp20 overexpression when compared to exosomes from WT CMs. Moreover, the Hsp20 contained in the exosomes was found to interact directly with Tsg101, an important factor needed for exosome biogenesis. On the one hand, the authors studied the paracrine effects of those exosomes under normal (NG) or hyperglycemic (HG) conditions in vitro using ECs. Thus, the exosomes isolated from cultured WT- and Hsp20-CMs were incubated with ECs in normal (NG) or high (HG) glucose conditions. In both, the Hsp20 exosomes increased the proliferation and angiogenesis potential of the ECs, with a more accentuated effect in ECs under HG conditions. On the other hand, autocrine effects, where CMs in NG or HG were used, revealed the same effects. Interestingly, the effects seen under HG conditions might be explained by the inhibition of oxidative stress. In fact, HG increased oxidative stress in ECs and CMs, which was attenuated by the exosomes from WT CMs, but was even more markedly attenuated when the cells were treated with the exosomes from Hsp20 CMs. Interestingly, the overall cardioprotective effects of overexpressing Hsp20 were impaired when the secretion of exosomes was inhibited. Finally, the authors demonstrated an in vivo cardioprotective effect of exosomes, showing that the administration of exosomes, WT, and Hsp20 was able to be uptaken by cardiac tissue and that the Hsp20 protein carried in the exosomes could be transferred to the heart. Interestingly, STZ-induced diabetic mice showed a decrease in cardiac apoptosis and increased angiogenesis when treated with Hsp20 exosomes [15].

Another study showing the transfer of EVs from CMs to ECs focused on understanding the potential cardioprotective effects of ticagrelor, a purinergic drug, in vitro. To this aim, EVs were derived from CMs (H9c2 cells) treated with ticagrelor and were given to ECs or CMs under HG, mimicking diabetic conditions. This in vitro model showed that the EVs from ticagrelor-treated CMs were able to (i) induce angiogenic capacity by modulating EC migration and tubulogenesis and (ii) reduce oxidative stress and apoptosis induced by hyperglycemic conditions in CMs. Moreover, some miRNAs are known to decrease in CMs under diabetic conditions, like *miR-499*, *-133a*, and *-133b*. In this in vitro model of DCM, it was also shown that EVs from ticagrelor-treated CMs had increased levels of these miRNAs, and, actually, these EVs could revert the levels of cellular miRNA in CMs under HG conditions. These results, therefore, reveal the cardioprotective effects of ticagrelor on DCM through the action of EVs [91].

Interestingly, many studies have revealed communication in the opposite direction from that described above; therefore, how exosomes are released from ECs has a direct implication on CM biology. For example, Davidson et al. demonstrated that healthy exosomes from non-diabetic subjects are able to have a cardioprotective effect. In order to obtain this conclusion, the authors used primary CMs subjected to hypoxia and reoxygenation to mimic cardiac injury and treated those cells with exosomes isolated from non-diabetic and diabetic rats and patients. The first observation was that the exosomes from non-diabetic subjects could rescue cell death in an injury model of CMs. Instead, the exosomes isolated from diabetic subjects were not able to reduce CM death after an insult. Since plasma exosomes originate from different sources, the authors used ECs to further understand their cardioprotective effect. Thus, the exosomes isolated from control ECs and ECs in hyperglycemia were used to treat CMs under stress conditions and, once again, the authors were able to observe protection against apoptosis only when the CMs were treated with exosomes from the EC controls, not from ECs in hyperglycemic conditions. Moreover, this study further demonstrated that exosomes from healthy rats were able to have a protective effect on CMs in diabetic rats. Since the authors previously showed that the Hsp70 on the surface of these exosomes is able to induce toll-like receptor 4 (Tlr4), leading to cardioprotective effects [92], they aimed to understand whether the Hsp70 present on the exosomal surface could be inhibited by the presence of hyperglycemia. Thus, the authors focused on the actual activation of kinase pathways through the exosomes, demonstrating that only the CMs treated with exosomes from healthy rats, and not in diabetic ones, were able to activate phosphorylation, demonstrating the direct role of the secreted exosomes in the activation of the kinase cascade, leading to the cardioprotective phenotype [93].

Autophagy and apoptosis are regulated, in part, by the Hippo pathway [94], where mammalian sterile 20-like kinase 1 (Mst1) is known to be an important element. In order to better characterize the effect of Mst1 in DCM, Hu et al. developed an in vivo model of diabetes where Mst1 was overexpressed specifically in ECs. In this model, the authors found worsening cardiac function and aggravated insulin resistance when Mst1 was overexpressed. In addition, increased levels of Mst1 protein were actually found in the exosomes derived from isolated cardiac ECs in these mice, and, interestingly, these exosomes were able to be uptaken by CMs, leading to decreased autophagy and increased apoptosis under high-glucose conditions [95].

Although ECs and CMCs are critical cell types during cardiac remodeling in DCM, and although we have just seen their potential impact on cellular communication by sending messages through EVs, it is obvious that other cell types may contribute to cell damage. Fibroblasts (FBs) have been identified as active cellular recipients during communication as they play an important role during myocardial fibrosis induced by DCM. For example, Zhao et al. observed the beneficial effects of isosorbide mononitrate, a drug used as a vascular dilator to improve cardiac function [96], in a model of DCM in rats. The authors studied the beneficial role of exosomes in DCM, showing that when these rats were treated with an inhibitor of exosomes (GW4869), the protective effect of the drug was suppressed. Interestingly, exosomes from the serum of DCM rats treated with isosorbide mononitrate were loaded with *miR-378*, *-30c*, *-208*, and *-21*. Specifically, the authors found that exosomal *miR-378* from DCM rats was able to suppress fibroblast proliferation when using cultured primary cardiac FBs by targeting the Igf1rG/Stat3 signaling pathway [97].

Immune cells, specifically macrophages (Mϕs), are infiltrated at the site of cardiac damage and mediate not only inflammation but also interactions with other cell types, such as FBs, promoting cardiac fibrosis [98]. The RNA-binding protein human antigen R (HuR), which regulates gene expression by altering mRNA stability or degradation, was found to increase in the CMs from HF patients. Furthermore, in diabetic mice, the HuR levels had increased in the cardiac tissue and Mϕs. Since HuR was associated with inflammation during CVD pathogenesis [99], the authors focused on better understanding the role of HuR in the cellular crosstalk mediated through EVs under diabetic conditions. Thus, exosomes were isolated from the in vitro cultures of Mϕs in high-glucose conditions and were used to treat FBs, which showed increased expression levels regarding the genes involved in inflammation and fibrogenesis. Moreover, when exosomes from HuR-silenced Mϕs were used, the effects were blunted. Finally, the authors demonstrated the role of HuR transferred from Mϕs to FBs through exosomes in cardiac fibrosis. Briefly, Angiotensin II-treated mice were injected with exosomes isolated from primary Mϕs from diabetic mice with or without HuR, observing that exosomes from diabetic animals induced fibrosis and cardiac dysfunction; however, the administration of HuR-deficient exosomes was cardioprotective by inhibiting cardiac inflammation and fibrosis [100]. Overall, these results showed that exosomes are able to transport the RNA-binding proteins secreted from Mϕs, which impact cardiac remodeling by modulating FB function.

Angiotensin II (AngII) type 1 receptor (AT1R) is a G protein-coupled receptor (GPCR) and is a key regulator of cardiac homeostasis and heart function by modulating blood pressure. Since GPCRs have been shown to be loaded into exosomes, and AT1R can be activated by biomechanical stresses, such as pressure overload, Pironti et al. investigated the function of At1r contained in the exosomes released under biomechanical forces as a communication protein to modulate cardiovascular function. To this aim, the authors mimicked mechanical overload in vitro by stimulating WT- or At1r-overexpressing HEK293T cells with osmotic stretch or with AngII, observing an increase in exosome release into the medium, which increased even further in At1r-overexpressing cells. Interestingly, the At1r contained in the exosomes could be transferred to other cells, maintaining their biochemical function. Indeed, the authors showed the in vitro cell-to-cell transfer of At1r-containing exosomes, activating agonist-dependent signaling. On the other hand, the in vivo induction of pressure overload by transaortic constriction (TAC) showed a three-fold increase in the exosomes released from the serum of TAC mice when compared to controls (sham). Even more interestingly, the authors showed that an in vivo injection of At1r-enriched exosomes (from hypotonic cells or from the serum of TAC mice) in AngII-challenged At1r KO animals was able to increase systolic blood pressure. In addition, hearts isolated from treated animals revealed the activation of the At1r signaling pathway. Finally, the study shows that injected At1r-enriched exosomes were able to be uptaken by troponin-positive CMs, identifying a function at the local level and skeletal muscle, functioning as distant communication molecules that were able to maintain cardiac homeostasis [101].

Fascinatingly, it is known that parasympathetic (located on the dorsal surface of the atria) and sympathetic ganglionic neurons (localized stellate ganglia) are involved in the cardiac autonomic function [102]. A study demonstrated the communication between primary ganglionic neurons and CMs (H9c2 rat cell line) by analyzing the effects of exosomes secreted from parasympathetic and sympathetic ganglionic neurons. Of note, this study shows that only the exosomes derived from the parasympathetic neurons had cardioprotective effects. In fact, the authors observed the inhibition of pro-apoptotic proteins in an in vitro model of hyperglycemia-induced apoptosis in CMs, as well as an amelioration of cell viability [103].

**Figure 2:**
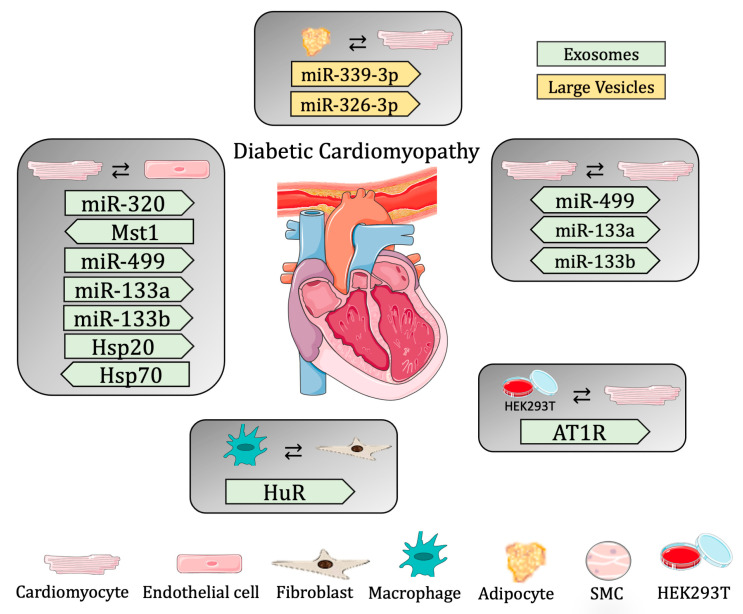
Summary of cell-to-cell communication in diabetic cardiomyopathy through extracellular vesicles.

Since hyperglycemia and oxidative stress are the main stress factors causing premature senescence, and the adipose tissue from diabetic patients displays senescent markers [104], Lin and colleagues demonstrated communication between adipose and cardiac tissue. In this study, the authors showed that the removal of the senescent adipose tissue from diabetic mice ameliorated cardiac function. In addition, the authors investigated the role of LEVs in DCM and showed that the LEVs isolated from the supernatant of cultured adipose tissue from diabetic and non-diabetic mice were able to be uptaken by CMs. Moreover, CMs treated with LEVs from diabetic animals had a worse cardiac function, presenting with slower contraction and decreased mitochondrial membrane potential, and increased mitochondrial superoxide (dysfunctional mitochondrial respiration). The authors also identified two miRNAs (*miR-339-3p* and *-326-3p*) overexpressed in the LEVs from diabetic mice that are responsible for the diastolic dysfunction of diabetic hearts [105] (Table 2 and Figure 2).

#### 3.2.3. Sepsis-Induced Cardiomyopathy

Traditionally, although sepsis-derived cardiac dysfunction was associated with circulating pro-inflammatory cytokines, several pieces of evidence suggest that exosome-mediated cell communication plays a central role in the onset and prognosis of sepsis. In this section, we review the current literature concerning cell-to-cell communication in different in vivo and in vitro models of sepsis [106].

It is known that a large number of extracellular particles, including pro-inflammatory cytokines and EVs, are released upon a septic shock, especially exosomes derived from bacterial-infected Mϕs, which are highly pro-inflammatory. Essandoh K et al. blocked the biogenesis/release of Mϕs-derived exosomes in septic mice, using GW4869 (sphingomyelinase inhibitor) to test whether this blockage could improve cardiac function after sepsis shock. Interestingly, GW4869 treatment reduced cardiac inflammation and increased survival in mice. This highlights that Mϕs-derived exosomes enhance cardiac inflammation and, thus, the prognosis of cardiac dysfunction, meaning that interrupting cell-cell communication has a therapeutic value [107].

Among all the disrupted regulatory mechanisms occurring during sepsis, increased apoptosis in various cell types is known to contribute to organ dysfunction, including the cardiovascular system [108]. Janiszewsk et al. demonstrated that, in patients, following a sepsis response, the levels of exosomes in the blood increased, especially apoptotic-derived particles from platelets containing Nicotinamide adenine dinucleotide phosphate (NADPH) oxidase subunits. In order to assess whether these exosomes lead to vascular malfunction, they isolated exosomes from sepsis patients and transferred them to cultured ECs and SMCs. Interestingly, they found that the oxidase capacity of exosomes increases ROS production in recipient cells, leading to apoptosis and, thus, vascular dysfunction [109]. Moreover, another study demonstrated that platelet-derived exosomes decreased myocardial contractility in isolated rabbit hearts. In fact, besides NADPH oxidase subunits, platelets also release NOS in exosomes that are uptaken by CMs to increase myocardial nitric oxide (NO) production, which has been associated with sepsis-induced myocardial dysfunction. Thus, they provided a novel mechanism independent from the known cytokine activation of myocardial NO, highlighting the role of carrier exosomes in response to sepsis-induced myocardial dysfunction [110]. Interestingly, Gambim et al. [111] demonstrated that the Nos contained in platelet-derived exosomes also plays an active role in vascular signaling. Briefly, aortic rabbit ECs were treated with platelet-derived exosomes from septic patients, helping to discover that exosomes could activate endothelial caspase-3, further promoting apoptosis by generating superoxide, NO, and peroxynitrite in the recipient vascular cells.

Recently, the role of exosomal *miR-126* shuttled from ECs to the myocardium in response to a septic shock was described. Specifically, endothelial heat shock protein family A (Hsp70) member 12B (Hspa12b) activates the shuttling of *miR-126*, which regulates adhesion molecules and immune cell infiltration in the myocardium, protecting the heart from septic cardiomyopathy. Hspa12b is mostly expressed in ECs, where it controls the expression of pro-migratory molecules, such as VEGF, playing an important role in angiogenesis. The knockdown of Hspa12b in HUVEC cells reduced migration capacity, wound-healing, and tube formation [112], while in vivo overexpression in mice showed improved protection against LPS-induced cardiac dysfunction. Moreover, *miR-126* has also been related to affecting EC adhesion by targeting the Vegfr2 pathway [113]. Interestingly, Hspa12b^−/−^ mice show low levels of *miR-126* upon LPS sepsis induction and suffer from aggravated cardiac dysfunction after sepsis induction. However, Hspa12b^−/−^ mice treated with *miR-126*-containing exosomes showed attenuated dysfunction by suppressing the expression of adhesion molecules and decreasing the infiltration of inflammatory cells into the myocardium. This work proposed that *miR-126* plays an important role in the Hspa12b regulation of adhesion molecule expression during sepsis; however, the mechanism remains elusive [114].

Recently, the role of *miR-885-5p*/HMBOX1 was discovered in a human cardiomyocyte cell line, AC16, treated with septic exosomes [115]. *miR-885-5p* was classically associated with cell growth, senescence, and/or apoptosis [116], while Homeobox-containing protein 1 (HMBOX1) inhibits the inflammatory response and regulates cell autophagy and apoptosis [117]. In silico analysis and further in vitro validation identified HMBOX1 as a downstream target of *miR-885-5p*. AC16 cells treated with the septic exosomes from patients revealed increased levels of *miR-885-5p* and decreased levels of HMBOX1, as well as the secretion of inflammatory cytokines that were further associated with increased pyroptosis-related proteins in hearts. Interestingly, the treatment of AC16 cells with *miR-885-5p* mimics was also able to induce an increase in secreted cytokines and pyroptosis proteins, an effect that was blunted when overexpressing HMBOX1. These results show the negative regulation of HMBOX1 by *miR-885-5p* and the significance of this axis in myocardial cell pyroptosis [115].

**Figure 3:**
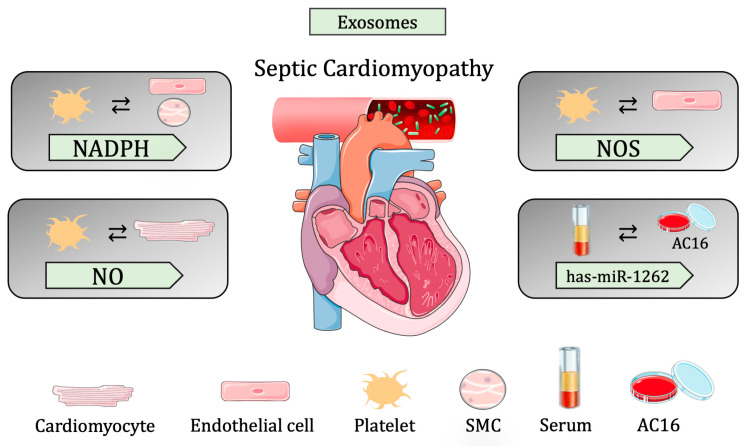
Summary of cell-to-cell communication in sepsis-induced cardiomyopathy through extracellular vesicles.

In line with these findings, *has-miR-1262* was found in the exosomes of septic patients and was shown to trigger SLC2A1 downregulation in AC16 cells. More specifically, the AC16 CMs exposed to exosomes from septic patients showed reduced glycolysis activity and increased apoptosis due to, in part, increased hsa-miR-1262 uptake and, therefore, decreased SLC2A1. To this end, the silencing of SLC2A1 promoted apoptosis and suppressed glycolysis, while the overexpression of SLC2A1 resulted in the opposite effect. They showed a novel signaling pathway that suggests suppression in CM proliferation via *hsa-mir-1262* and its target SLC2A1 [118] (Table 2 and Figure 3).

### 3.3. Extracellular Vesicles Used as Carriers

Over the last few decades, a plethora of synthetic nanoparticle delivery systems have emerged and have been utilized in order to ameliorate the pharmacokinetic and pharmacodynamic profile of therapeutics, as well as reduce drug toxicity and off-target side effects [119]. So far, improved delivery has been obtained through the optimization of the size, shape, and surface properties of nanocarriers, and liposome-based nanoparticles are among the most prominent examples of these synthetic drug delivery systems in the clinic in terms of FDA approvals [120,121,122,123]. However, the past few decades have witnessed a limited number of approved liposome-based nanoformulations, such as Doxil, Mepact [124,125], and Abraxane [126]. Indeed, their successful clinical translation is still hampered by some limitations, such as (i) the complex interactions between nanoparticles and the biological environment, including the formation of a protein corona that mask surface ligands and triggers immunological recognition, (ii) rapid clearance from the bloodstream, (iii) off-target accumulation in filtering organs, and (iv) triggering the innate immune response [127]. In this context, EVs have just recently entered this field as biological alternatives for the delivery of therapeutics with the hope to overcome the limitations of liposomes. EVs, as phospholipid bilayer-encapsulated vesicles, can also be considered natural drug delivery carriers [128], and one of the main attractive advantages of EVs as drug delivery systems (DDSs) over synthetic drug delivery reagents is their innate limited immunogenicity and cytotoxicity. As a matter of fact, synthetic lipid nanoparticles always induce a toxic immune response in vivo and do not perform as well as predicted [129]. On the contrary, due to their endogenous origin, surface composition, and high biocompatibility, EVs have been demonstrated to be less challenged by the immune system [130]. Moreover, another advantage of EVs is their high capacity to be uptaken by target cells. Indeed, recent studies suggest that EVs can transfer between 10% and 30% of the RNA cargo to the cytosol of recipient cells; in contrast, lipid nanoparticle systems are estimated to deliver only ~1–2% [131,132]. Additionally, another advantage of EVs is their potential ability to cross biological barriers, such as the blood-brain barrier, which is notoriously difficult to breach using synthetic drug delivery vehicles [133]. However, despite promising targets, EVs exhibit some limitations that hamper their effective use in the clinical field, such as low yield, insufficient consistency between batches, and insufficient targeting ability and circulation stability [134]. Therefore, appropriate protocols need to be established in order to improve strategies for source selection, cargo loading, and surface modification to develop more optimized EV-based DDSs. In light of these limitations, recent studies have focused efforts on the improvement of EV loading for use as therapeutic molecules. One such study outlined the use of a novel technology, Exo-Load, which takes advantage of microfluidics for drug loading into exosomes [135]. The use of microfluidics can be exploited in the context of the drug loading of EVs in order to ameliorate the efficiency of EVs as carriers of various molecules necessary for therapeutic targeting.

#### 3.3.1. Myocardial Infarction

The use of EVs has been gaining a lot of popularity as a means of cell-based therapy in heart failure. The injection of loaded EVs has currently been used through direct injection to the damaged myocardium, although this method currently presents many limitations regarding the efficacy of the treatment. In order to bypass the current limitations, a recent study exploited the delivery of EVs loaded into a clinical-grade hyalonic acid (HA) biomaterial. Using EVs from umbilical cord-derived mesenchymal stromal cells (HUCMSC), this study showed that not only were the HA-embedded EVs sustained in the myocardium for more than 10 days, but they improved angiogenesis, decreased apoptosis and fibrosis, and maintained cardiac function. The use of biomaterials to optimize the efficiency of EV targeting regarding the damaged myocardium presents an exciting new direction for cell-based therapies post-MI [136].

The use of cardiac progenitor cell-derived EVs (CPC-EVs) has also been an exciting target for cell-therapy-based treatments of MI. CPC-EVs have been shown to be cardioprotective through the delivery of endoglin, which promotes angiogenesis [87]. Moreover, CPC-EVs could also carry *miR-146a-3p*, *miR-132*, and *miR-201*, which are known to be pro-angiogenic [137]. Furthermore, a recent study, therefore, described the use of coupling biomaterial delivery vehicles to maximize the delivery and potential of CPC-EVs. In this study, the authors developed a bioengineered strategy for cardiac repair using biomimetic microcarriers to maximize CPC therapeutic properties. This study demonstrated that biomimetic microparticles allow for higher CPC retention in the infarct area in chronic MI, followed by a subsequent increase in cardiac function, ventricular remodeling, and angiogenesis. Moreover, they demonstrated that the EVs from CPCs play a role in cardiac remodeling post-MI by promoting an anti-fibrotic phenotype. Therefore, the use of biomimetic microcarriers can be used to enhance the effects of EVs, thus improving the currently available cell therapy strategies [138]. Similarly, the EVs isolated from mouse FBs-derived iPSC were shown to be enriched in the miRNAs associated with angiogenesis, thus rendering the recipient cells with a higher adaptation capacity to hypoxic stress. More specifically, these FB-iPSC-EVs were shown to carry *let-7*, *miR-145*, the *miR-17*–*92* cluster, and *miR-302a-5p*, miRNAs which have been shown to be involved in cell-survival-related pathways [139]. Moreover, it was shown that hypoxic EVs derived from hESC-CVPs contain high levels of lncRNA *MALAT1*, which promotes angiogenesis and cell viability through a miR-497-dependent mechanism [140].

As previously mentioned, MI results in irreversible damage to the myocardium as a result of ischemia and necrosis. Since CMs are not able to regenerate, prolonged exposure to a hypoxic environment and damage to the myocardium eventually result in heart failure. The ischemic response involves the activation of inflammatory Mϕs, which accumulate to remove necrotic cells. Activated FBs then migrate to the site of injury, resulting in fibrosis and decreased cardiac function. In order to prevent CM damage post-MI, the use of EVs as carriers has been exploited with respect to the inflammatory response. Indeed, a study using bone marrow-derived EVs showed that they are able to maintain an anti-inflammatory phenotype through *miR-182*, which promotes cardiac repair through the inhibition of TLR4 in the recipient cells [141]. Another similar study using adipose tissue-derived MSCs showed that EVs derived from this tissue possess cardioprotective properties, specifically by activating anti-inflammatory pathways, reducing the serum levels of Il-6, Il-1β, Tnf-α, and Ifn-γ, and improving cardiac function. Adipose tissue-derived MSCs were also shown to have the capacity to inhibit myocardial infarct expansion and apoptosis through the regulation of the Wnt/β-catenin signaling pathway post-MI [142]. HUCMSC-EVs have also been shown to have anti-inflammatory properties. In fact, a recent study utilized HUCMSC-EVs, showing their anti-inflammatory properties by decreasing the expression of *miR-125b-5p*, a miRNA that is activated post-MI, and through the activation of Smad7 expression [143].

Many studies have revealed the cardioprotective effects of exosomes. To this end, a recent study showed that ESC-derived exosomes carry *miR-294*, and the delivery of these exosomes to the infarct area promoted angiogenesis and cell survival, thus improving cardiac function [144]. Similarly, it was shown that human pericardial fluid-derived exosomes carry *let-7b-5p*, targeting the TGFBR1 gene. The delivery of *let-7b-5p* to ECs via exosomes promoted angiogenesis and promoted cardiac repair, as mentioned above [145]. Exosomes carrying *miR-126* and *miR-146a* delivered to the infarct site in a rat model of MI showed that these miRNAs promoted the cardiac regeneration of the damaged myocardium through the regulation of angiogenesis, cell migration, tube formation, and vascular endothelial growth factor folding. Interestingly, exosomes loaded with these miRNAs and further encapsulated in an alginate hydrogel, resulted in reduced infarct size and improved angiogenesis post-MI [146]. Moreover, exosomes isolated from MSC resulted in the partial restoration of cardiac function and reduced Ezh2 expression in the myocardium of rats post-MI. Conversely, this study showed that Ezh2 inhibited the effect of MSC-EXO on the recovery of cardiac function and accelerated fibrosis, providing yet another piece of evidence of cellular crosstalk and communication through EVs [147]. Interestingly, it has been shown that during acute myocardial infarct, exosomes alter their content in order to activate cardioprotective mechanisms in the damaged myocardium. For example, *miR-214*, which inhibits the expression of the Na^+^/Ca^2+^ exchanger 1, the increased regulation of cell death was found in exosomes post-MI, regulating calcium overload and, in turn, promoting a protective effect of CMs and maintaining cardiac contractility and function [3]. Another interesting study utilizing exosomes as a cell-based therapy revealed a novel approach for enhancing exosome uptake by cardiomyocytes. The overexpression of exosomal Cxcr4 increased the efficiency of plasmatic injections of exosomes of cardiac resident progenitor cells to ischemic hearts, promoting a cardioprotective effect. Moreover, Exo-Cxcr4 injection significantly reduced infarct size and improved cardiac function post-MI. This provides another important advancement in our knowledge for the more efficient targeting of exosomes to the infarct site [148]. Finally, the use of intra-pericardially injected exosomes for MI treatment was described, whereby they evaluated inflammation and cardiac repair. This study revealed a novel mechanism regulating MSC exosomes through the Pp2a/p-Akt/Foxo3 signaling pathway, modulating the immune response post-MI via intrapericardial injection and stimulating cardiac repair [149]. Similarly, the use of cardiosphere-derived cells (CDCs) has been exploited for therapeutic purposes, as these cells have the ability to secrete exosomes and EVs that possess anti-inflammatory, anti-fibrotic, angiogenic, and cardiomyogenic properties. The benefits of using a cell-based therapy for the functional improvement of cardiac function post-MI have been widely described [146,150].

On the other hand, a recent study proposed the use of engineered small-EVs, (sEV)-like vesicles (ELV), using synthetic mimics as the cargo in order to provide an alternative method for cell therapy, taking advantage of the inherent properties of EVs. Interestingly, the authors were able to formulate ELVs derived from cardiomyocyte progenitor cell EVs containing *miR-126*. The internalization of these particles promoted angiogenesis, providing yet another example of a site-directed cardiac repair post-MI. Moreover, loading such particles with synthetic cargo, such as mimics of miRNA, can provide a more efficient therapeutic tool for repair and regeneration [6].

Aside from miRNAs, many studies have reported the loading of long noncoding RNAs into EVs, which regulate cell proliferation, migration, angiogenesis, and immune responses in hypoxic conditions post-MI. It has been shown that the lncRNAs *UCA1*, *MALAT1*, *NEAT1*, *KLF3-AS1*, and HCP5 transported via EVs promote CM survival and improve cardiac function through the inhibition of CM autophagy via *miR-143*, *miR-92a*, *miR-23c*, *miR138-5p*, and *miR-497*. On the contrary, EV-associated lncRNA *HCG15*, *miR-153-3p*, and *miR-328-3p* were found to exacerbate ischemic injury post-infarct via NFκB/p65, p38, PI3K/Akt, and caspase-3-dependent mechanisms. Therefore, understanding the content of EVs will be crucial for targeting cardiomyocyte survival mechanisms post-MI [55] (Table 3).

#### 3.3.2. Diabetic Cardiomyopathy

MSCs are pluripotent stem cells that have been involved in different pathologies, including diabetes, where MSC therapy has been considered as a treatment. However, MSCs have limitations, including tumorigenesis and a low survival rate. Since MSCs are known to secrete several soluble factors, several studies discovered the potential of MSC-EXO, which has been shown to behave as effectively as MSCs as therapeutic tools themselves or to be used as carriers for different molecules or drugs. An example of the use of MSC-EXO in DCM comes from a study in which researchers used a rat model of diabetes: it was observed that treatment with MSC-EXO was able to ameliorate myocardial injury. MSC-EXO was capable of reverting cardiac alteration induced by diabetes, such as reducing left ventricle collagen and increasing the enzymes involved in lipid metabolism. Moreover, the low protein levels of TGF-β1 and Smad2 indicate that MSC-EXO may be cardioprotective by blocking myocardial injury and fibrosis through the inhibition of the TGF-β1/Smad2 pathway [151].

Another study showed the role of HUCMSC-EXO in controlling autophagy in DCM. Autophagy is a form of cell death that arises in several cells from the cardiovascular system, including CMs, ECs, SMCs, and Mϕs, and is critical for maintaining cellular homeostasis [152]. Autophagy is the response of CMs against several cellular stresses, including high glucose levels caused by diabetes [153]. However, dysregulated autophagy may lead to cardiac apoptosis and fibrosis, which may cause heart failure. Indeed, DCM patients present aberrant levels of autophagy. Zhang et al. studied the potential benefit of HUCMSC-EXO as a therapeutic strategy to target and ameliorate DCM using a rat model. The authors showed that DCM rats treated with HUCMSC-EXO had beneficial effects on cardiac function and were able to attenuate myocardial autophagy by regulating the AMPK-ULK1 signaling pathway [154] (Table 3).

#### 3.3.3. Sepsis-Induced Cardiomyopathy

In line with the previous section, cellular treatments using MSCs were demonstrated to reduce mortality and attenuate heart failure during sepsis in mice. Briefly, MSCs interact with circulating Mϕs and other tissues to reduce the secretion of pro-inflammatory cytokines; however, after MSC infusion, they migrate primarily to the lungs and kidney [155]. This led Wang et al. to hypothesize that there could be indirect communication, through exosomes, between MSCs and CMs. In fact, they found that the miR-233 released from MSCs has a protective effect on sepsis-induced myocardial dysfunction. miR-233 intake in CMs downregulates the expression of Sema3A and Stat3, leading to a reduction in the inflammatory response and cell death. Thus, septic mice treated with exosomes derived from MSCs-miR-223-KO have reduced cardiac function, aggravated inflammatory responses, and higher mortality in contrast to WT-MSCs. This work presented the basis of a novel cell-free treatment to protect against heart failure induced by sepsis.

Another example of the potential application of EVs as novel DDS is described in the work of Sun D et al. [156], where a cardioprotective natural compound, curcumin, when self-assembled into the lipid bilayer of EVs, was demonstrated to elicit better anti-inflammatory and anti-fibrotic effects. Indeed, the authors demonstrated that once encapsulated into exosomes, curcumin exhibited better aqueous solubility and stability and, in turn, resulted in a higher concentration in the blood and, therefore, an increased bioavailability. Moreover, the therapeutic relevance of this EV-embedded compound was confirmed in a mouse model of LPS-induced septic shock, in which the authors showed that animals treated with curcumin-EVs had significantly reduced levels of inflammatory factors (Table 3).

## 4. Discussion and Future Directions

The present review extensively summarizes three very different pathologies of the cardiovascular system: myocardial infarction (MI), diabetic cardiomyopathy (DCM), and sepsis-induced cardiomyopathy (SIC). In these three cases, we focused on analyzing in detail the literature regarding the role of extracellular vesicles (EVs) from three points of view: (1) the identification of EVs as biomarkers, (2) cell-to-cell communication through EVs as vehicles for cellular information transfer, and (3) the use of EVs as carriers themselves or harboring specific molecules.

Extracellular vesicles have become an increasingly promising prognostic and therapeutic tool in cardiovascular diseases, as they are known to carry biological information necessary for cell-to-cell communication. Moreover, the use of EVs has been shown to be an important therapeutic tool for MI therapy and cardiac repair. Although many studies have demonstrated increased EV secretion post-MI, very little is known about EVs as potential biomarkers in less-studied pathologies such as DCM or SIC. Since EVs carry various cargos, ranging from proteins to ncRNAs, they have been widely used as biomarkers to assign specific molecules to specific stages of a pathology or treatment: this has allowed for a significant improvement in the detection of a pathology, evaluation of the progression of a pathology and in following the response to therapy [40]. Therefore, it is crucial to identify different levels and content in EVs to help increase the development of novel therapeutic approaches. Interestingly, microfluidics systems have been proposed for the isolation, characterization, and analysis of EVs [135,157].

As explained, aside from their role as biomarkers, EVs have been identified as transport molecules for proteins or noncoding RNAs between different cell types involved in CVDs, thus playing an important role as communication molecules for homeostasis maintenance. In the case of identifying EVs directly functioning as cell-to-cell communication molecules, several studies have been published in MI, DCM, and SIC. The main cell types involved during cellular communication include CMCs, ECs, FBs, and Mϕs. However, other cell types have been discovered as having a direct impact on cellular communication, such as cells from the adipose and nervous tissues in DCM. Finally, we focused on EVs which, when exploited as carriers, are able to ameliorate myocardial function in the above-mentioned pathologies. Although very little is known about this topic in DCM and SIC, the use of mesenchymal stem cell-derived exosomes (MSC-EXOs) are being exploited as potential therapeutic tools themselves or used as carriers for different molecules or drugs [158]. Interestingly, MSCs derived from human iPSC (iPSC-MSC) have been shown to be an alternative cell source of MSCs, demonstrating a better proliferation rate, survival and therapeutic efficacy compared to bone marrow-derived MSCs [157,159,160,161]. Of note, iPSC-MSC clinical trials showed large-scale production, safety, and efficacy [162]. Thus, molecules secreted from iPSC-MSC, such as EVs, might be advantageous for therapeutic approaches. Therefore, although current treatments for MI include cardiac reperfusion, antithrombotic therapy, and angioplasty [163], such interventions result in further damage of the myocardium, and more effective therapeutic strategies are necessary. Utilizing the inherent molecular pathways, namely release of extracellular vesicles activated by aberrant external signals in order to maintain CM homeostasis, will be a key therapeutic strategy for exploiting new avenues in cell therapy and regenerative medicine, by promoting CM survival and regeneration. MSC-derived EVs have become an attractive direction of study, specifically regarding their potential as therapeutic targets, given their low-immunogenicity. Interestingly, they have been shown to prompt tissue regeneration and play an important role in immunomodulation [164]. In an elegant review by Moises de Matos et al., the role of pluripotent stem cell-derived EVs (PSC-EVs) in therapeutic applications was highlighted, noting the fact although MSC-EVs have been widely studied in various disease contexts, have many limiting factors for their use stemming from the properties of MSCs as a source of EVs. These include EV variability derived from different tissues, limited ability of MSCs to proliferate and their genomic instability after a few passages. Instead, PSC-EVs have been implicated as having protective effects in vivo and in vitro in various animal models of ischemia-reperfusion kidney injury, protection against ischemic stroke, and improvement in angiogenesis, to name a few. Moreover, this review highlighted the importance of PSC-EVs in their capacity to rejuvenate various cell types, including senescent endothelial cells, senescent human dermal fibroblasts, etc. [165]. This provides a clear example of the use of PSC-EVs in disease context and may provide, with many future applications in the context of cellular repair and regeneration, specifically with respect to CVDs and injury to the myocardium.

In diabetic cardiomyopathy, cardiac problems arise from the damaged microvasculature due to the effects of diabetes [166]. Moreover, heart failure in diabetic patients is difficult to detect since it occurs without any other comorbidity. Therefore, the identification of EVs in diabetic patients, which further undergo ventricular problems would be of critical importance to pinpoint potential biomarkers useful for early detection, avoiding potential cardiac complications during diabetes, as well as the identification of potential therapeutic targets able to ameliorate DCM. EVs play an important role as communication molecules also in DCM. Indeed, EVs have been identified to transport different kinds of molecules (proteins or miRNAs) between different cell types involved in the disease. Finally, we have discussed the contribution of EVs as vehicles for specific therapeutic approaches in DCM, understanding the lack of studies in this specific context.

In SIC, understanding the mechanisms of cell-to-cell communication is crucial for the prognosis of this disease. In this context, a plethora of studies on murine sepsis models and patients have demonstrated that the level of circulating EVs is altered compared to healthy controls. Despite the numerous functions of EVs that have been documented, such mechanisms remain largely unknown during sepsis and SIC pathology [167]. Although the heart is a multicellular organ whereby cell-to-cell communication plays a central role in the onset of several CVDs, there is an obvious lack of scientific knowledge about EV regulation and mechanisms in septic cardiomyopathy. This lack of knowledge slows the development of new therapeutics against sepsis, as current treatments are based only on the use of antibiotics, which tackle the infection source rather than the symptoms, but do not avoid the high mortality rate in septic patients [168]. Moreover, clinical studies using anti-inflammatory drugs also failed in reducing mortality [169], providing an ulterior motivation for understanding the molecular mechanisms of EV-mediated cell-to-cell communication in sepsis cardiomyopathy.

Finally, although nanomaterial science has progressed in the field of therapeutics by exploiting natural mechanisms of extracellular vesicle cell-to-cell communication, the use of EVs for the development of novel therapeutic strategies for CVDs is still in its infancy but provides a paramount opportunity for future pharmacological therapies, diagnosis, and prognosis.

Overall, given the vast literature on CVDs and EVs, which has become a very attractive field of study in the last decade, prevalently with respect to MI, this review highlights such mechanisms, providing common proteins, noncoding RNAs and communication mechanisms that can be applicable to DCM and SIC, two CVDs that much less represented in the literature.

## Figures and Tables

**Table 1:** Extracellular vesicles used as biomarkers in myocardial infarction, diabetic cardiomyopathy, and sepsis-induced cardiomyopathy.

Myocardial Infarction			
Cargo	Origin	EV Classification	Biological Function/Correlation
not specified	plasma	increase endothelial-derived EVs	increased mortality in HF patients
lncRNA Neat1	conditioned medium CMs	increased in EVs	CMs and FBs survival
miR-126	plasma	reduced in EVs	high-risk of CVD
miR-192, miR-194, miR-34a	plasma	increased in EVs	correlate with acute MI
miR-1915-3p, miR-457, miR-3656	serum	decreased in EVs	correlate with MI
miR-1, miR-208	serum and urine	increased in exosomes from damaged myocardium	correlate with post-acute MI
miR-130a	plasma	increased in EVs	attenuation of cardiac remodeling post-MI
miR-30a	serum and CMs conditioned medium	increased in exosomes	correlated with AMI and regulation of hypoxic response
miR-340, miR-424/miR-29b	serum	decreased/increased in EVs	correlate with MI
CD172a	plasma	increased in cardiac EVs	correlated in hypoxia conditions, as MI
HIF-1α and TGF-β	FB	increase exosome	increased vascular expression of collagens and fibronectin
APOD, APOC3, C1Q1A, C5, GP1BA, PPBP	plasma	EVs	predictive of MI and myocardial damage
not specified	plasma	platelet- and leukocyte-derived EVs	decreased during P2Y12 treatment
ceramides, dihydroceramides, sphingomyelins	plasma	increased in EVs	correlation with MI
**Diabetic Cardiomyopathy**			
**Cargo**	**Origin**	**EV classification**	**Biological function/correlation**
miR-1 and miR-133	CMs conditioned medium	increased in exosomes	correlation with lipid accumulation in CMs, diabetic model
miR-30d-5p and miR-126-5p	plasma	reduction in exosomes	correlation with HFpEF in diabetic rats
**Sepsis-induced cardiomyopathy**			
**Cargo**	**Origin**	**EV classification**	**Biological function/correlation**
miR-150-5p	neutrophils	decreased in EVs	contributes to the worsening of SIC
troponin I and muscle-associated glycogen phosphorylase	blood	CM-derived small- and medium EVs	LPS-induced systemic inflammatory response syndrome

**Table 2:** Extracellular vesicles used as cellular communication molecules in myocardial infarction, diabetic cardiomyopathy, and sepsis-induced cardiomyopathy.

Myocardial Infarction				
Cargo	Donor Cell	Recipient Cell	EV Classification	Biological Conditions
TNF-α	CMs	CMs	exosomes	induced by hypoxia in vitro, promotes inflammation
miR-126 and miR-210	ECs	CPCs	exosomes	under hypoxia, increasing cardiac progenitor cells resistance to hypoxic stress
ENSMUST00000122745/Neat1	CMs	FBs	small/large EVs	regulation FB survival, under hypoxic conditions
circ_0001747	adipose-derived stem cells	CMs	exosomes	protective effects against H/R
miR-195	CMs	FBs	exosomes	maintenance of cardiac homeostasis
miR-144	plasma	myocardium	EVs	cardioprotection, promoting cell survival during reperfusion
miR-133	EPC	FBs	EVs	regulation of cardiac fibrosis under hypoxia
circRNA Whsc1	CMs	ECs	EVs	cardiac regeneration post-MI
miR-222 and miR-143	CMs	ECs	EVs	protection against oxidative stress by enhancing angiogenesis
Hsp20	CMs	ECs	exosomes	cardioprotection by myocardial angiogenesis
miR-31	ASC	ECs	exosomes	enhance angiogenesis during ischemia
miR-30a	CMs	not specified	exosomes	regulate autophagy in a paracrine way, myocardium protection after MI
miR-21	Mesenchymal stromal cells	CMs and ECs	exosomes	INFg treatment, improved cardiac function in MI conditions
miR-30a, miR-100, miR-27a, and miR-30e	epicardial	CMs	exosomes	enhance proliferation of CMs in vitro and in vivo
not specified	cardiac progenitor cells	infarct side	EVs	CPCs transplantation enhances cardiac recovery post-MI
miR-155	immune cells	FBs and ECs	exosomes	repressing FB proliferation and EC angiogenesis
miR-126	serum	ECs	exosomes	promote angiogenesis in AMI patients
**Diabetic Cardiomyopathy**				
**Cargo**	**Donor cell**	**Recipient cell**	**EV classification**	**Biological Conditions**
miR-320 (up) and miR-126 (down)	CMs	ECs	exosomes	impairment of myocardial angiogenesis in vitro
Hsp20	CMs overexpressing Hsp20	ECs and CMs	exosomes	cardioprotective and increase angiogenesis in vitro and in vivo
miR-499, miR-133a and miR-133b	CMs	ECs and CMs	EVs	cardioprotective effects after ticagrelor treatment in vitro
Hsp70	ECs	CMs	exosomes	cardioprotective effects of exo-derived from healthy subjects, not diabetic
Mst1	ECs overexpressing Mst1	CM	exosomes	worsening of cardiac function and aggravated insulin resistance, in vitro and in vivo
miR-378	serum	FBs	exosomes	inhibition of FBs proliferation in DCM rats treated with isosorbide mononitrate
HuR (down)	MOs	FB	exosomes	increase expression of inflammatory genes and fibrogenesis, in vitro and in vivo
AT1R	serum or hypotonic cells overexpressing AT1R	CMs	exosomes	increase systolic blood pressure, maintain cardiac homeostasis
not specified	parasympathetic ganglionic neurons	CMs	exosomes	cardioprotective effect in vitro
miR-339-3p and-326-3p	adipose tissue	CMs	LEV	worsen cardiac function when diabetic exosomes are used
**Sepsis-induced cardiomyopathy**				
**Cargo**	**Donor cell**	**Recipient cell**	**EV classification**	**Biological Conditions**
not specified	MOs		exosomes	enhance cardiac inflammation
NADPH	Platelets	ECs and SMCs	exosomes	vascular dysfunction by increases apoptosis
NO	Platelets	CMs	exosomes	reduced myocardial contractility
NOS	Platelets	ECs	exosomes	increased ROS production
miR-126	ECs	Myocardium	exosomes	regulation of adhesion molecules and immune cell infiltration
not specified	Serum	CMs	exosomes	increases secreted cytokines and pyroptosis proteins
has-miR-1262	Serum	CMs	exosomes	reduces glycolysis activity and increased apoptosis

**Table 3:** Extracellular vesicles used as carriers in myocardial infarction, diabetic cardiomyopathy, and sepsis-induced cardiomyopathy.

Myocardial Infarction			
Cargo	Origin	EV Classification	Therapeutic Outcome
not specified	HUCMSC	HA-embedded EVs	improved angiogenesis, decreased apoptosis and fibrosis and maintained cardiac function
endoglin	cardiac progenitor cell	ECs	promoting angiogenesis
miR-146a-3p, miR-132 and miR-201	cardiac progenitor cell	EVs	cardioprotective by promoting angiogenesis
not specified	cardiac progenitor cell	biomimetic EVs	higher CPC retention in the infarct area in chronic MI, increasing cardiac function
let-7, miR-145, miR-17–92 cluster, and miR-302a-5p	mouse fibroblast-derived iPSC	EVs	improved cardiac repair induction of angiogenesis, adaptation capacity to hypoxic stress
lncRNA MALAT1	hESC-CVPs	EVs	angiogenesis and cell viability through a miR-497-dependent mechanism
miR-182	bone marrow	EVs	improving cardiac repair through inhibition of TLR4
not specified	adipose tissue-derived MSC	EVs	improving cardiac function, reducing serum levels of IL-6, IL-1β, TNF-α, and IFN-γ
not specified	HUCMSC	EVs	anti-inflammatory properties post-MI
miR-294	ESC-derived	exosomes	angiogenesis and cell survival
let-7b-5p	human pericardial fluid-derived	exosomes	promoted angiogenesis and promoted cardiac repair
miR-126 and miR-146a	-	exosomes encapsulated in alginate hydrogel	promoting cardiac repair
not specified	MSC	exosomes	partial restoration of cardiac function targeting EZH2
miR-214	bone-marrow	exosomes	regulate calcium overload
CXCR4	-	exosomes	cardioprotective effect
not specified	MSC	exosomes	intrapericardial injection and stimulating cardiac repair
not specified	cardiospheres	exosomes and EVs	anti-inflammatory, anti-fibrotic, angiogenic and cardiomyogenic properties
miR-126	cardiomyocyte progenitor cell	engineered small-EVs (sEV)-like vesicles (ELV)	cardiac repair post-MI by promoting angiogenesis
lncRNAs UCA1, MALAT1, NEAT1, KLF3-AS1 and HCP5	-	EVs	inhibition of cardiomyocyte autophagy
lncRNA HCG15, miR-153-3p and miR-328-3p	-	EVs	exacerbate ischemic injury post-infarct
**Diabetic Cardiomyopathy**			
**Cargo**	**Origin**	**EV classification**	**Therapeutic outcome**
not specified	MSC	exosomes	cardioprotective by blocking myocardial injury and fibrosis
not specified	HUCMSC	exosomes	beneficial cardiac function by attenuating myocardial autophagy
**Sepsis-induced cardiomyopathy**			
**Cargo**	**Origin**	**EV classification**	**Therapeutic outcome**
miR-233	MSC	exosomes	CMs uptake, reduction of inflammatory response and cell death
curcumin	self-assembled into the lipid bilayer	exosomes	anti-inflammatory and anti-fibrotic

## Data Availability

Not applicable.
